# Understanding intersectional inequality in access to primary care providers using multilevel analysis of individual heterogeneity and discriminatory accuracy

**DOI:** 10.1371/journal.pone.0296657

**Published:** 2024-01-19

**Authors:** Jennifer W. He, Amanda L. Terry, Dan Lizotte, Greta Bauer, Bridget L. Ryan

**Affiliations:** 1 Department of Epidemiology and Biostatistics, Western University, London, Ontario, Canada; 2 Schulich School of Medicine & Dentistry, Western University, London, Ontario, Canada; 3 Department of Family Medicine, Western University, London, Ontario, Canada; 4 Department of Computer Science, Western University, London, Ontario, Canada; 5 Institute for Sexual and Gender Health, University of Minnesota Medical School, Minneapolis, Minnesota, United States of America; UCL: University College London, UNITED KINGDOM

## Abstract

**Background:**

Despite the Canadian healthcare system’s commitment to equity, evidence for disparate access to primary care (PC) providers exists across individual social identities/positions. Intersectionality allows us to reflect the realities of how social power shapes healthcare experiences at an individual’s interdependent and intersecting social identities/positions. The objectives of this study were to determine: (1) the extent to which intersections can be used classify those who had/did not have a PC provider; (2) the degree to which each social identity/position contributes to the ability to classify individuals as having a PC provider; and (3) predicted probabilities of having a PC provider for each intersection.

**Methods and findings:**

Using national cross-sectional data from 241,445 individuals in Canada aged ≥18, we constructed 320 intersections along the dimensions of gender, age, immigration status, race, and income to examine the outcome of whether one had a PC provider. Multilevel analysis of individual heterogeneity and discriminatory accuracy, a multi-level model using individual-level data, was employed to address intersectional objectives. An intra-class correlation coefficient (ICC) of 23% (95%CI: 21–26%) suggests that these intersections could, to a very good extent, explain individual variation in the outcome, with age playing the largest role. Not all between-intersection variance in this outcome could be explained by additive effects of dimensions (remaining ICC: 6%; 95%CI: 2–16%). The highest intersectional predicted probability existed for established immigrant, older South Asian women with high income. The lowest intersectional predicted probability existed for recently immigrated, young, Black men with low income.

**Conclusions:**

Despite a “universal” healthcare system, our analysis demonstrated a substantial amount of inequity in primary care across intersections of gender, age, immigration status, race, and income.

## Introduction

Globally, primary care functions to deliver “promotive, protective, preventive, curative, rehabilitative, and palliative services throughout the life course” [1]. Stronger primary care systems are associated with higher satisfaction in health services, better health (based on indicators such as low birthweight ratio, total infant mortality, age-adjusted life expectancy, years of potential life lost), lower health care costs, and lower levels of medication use [2–4]. In Canada, primary care serves as a gatekeeper to the health care system, regulating access to specialist care and other health care services [5]. Thus, a criterion of the *Canada Health Act* is universality, which entitles all residents of a province (with some exceptions) to the publicly insured health care services of that province, on uniform terms and conditions [6].

The goal of primary care is not only to provide care to all, but to address the needs of those who are most vulnerable and who confront additional challenges to remaining healthy [7]. This commitment to equity is reflected in the *Canada Health Act*, under which accessibility to insured services provided by the provinces and territories (e.g., unhindered by financial charges and discrimination based on health status, age, or financial circumstance) is one of the principles [6]. Despite this, evidence for inequalities in indicators of access to primary care persists, particularly for immigrants [8–11] and those with low income [10].

Andersen and Newman’s Framework of Health Service Utilization is a well-established conceptual framework to examine factors that contribute to an individual’s utilization of health care [12]. These factors are grouped into three interrelated categories. Predisposing factors are pre-existing factors that may predict propensity to use health care services, including age, sex, race, and other demographic and social structural elements. Enabling factors are family and community-related resources available for seeking care, such as income or rurality of region. Illness level/need factors refer to the existence or anticipation of an individual’s illness. Importantly, these factors do not always fit exclusively under one of the three categories [13]. For instance, immigration status is a predisposing factor but can also enable (or prevent) one from utilizing a health service due to availability of resources and ability to access resources. Although Andersen and Newman’s framework encompasses a diversity of factors, research applying it to study the social determinants of health outcomes and access to health care is conventionally performed by theorizing and analyzing factors as independent. Individuals are assumed either to primarily experience a single factor or that their experiences exist “in parallel” and can be captured simply by obtaining the sum of the effects of each individual factor. In reality, individuals often experience a unique set of stressors and barriers that are related to their membership in multiple social groups and the power relations within these groups in a particular context [14].

To address research challenges posed by the theoretical and mathematical non-additivity of an individual’s experiences, researchers have drawn on intersectionality theory. Intersectionality is a theoretical framework used to conceptualize, understand, and act on complex social inequalities that emerge from interlocking social identities/positions and power relations between these identities/positions [15]. The core ideas of intersectionality were explored by women of color in American social movements throughout the 1960s and 1970s [16]. The term “intersectionality” was later coined and introduced into the academy by legal scholar Crenshaw in her seminal papers [17, 18]. Crenshaw discussed how the overlapping subordinate positions that women of color occupy shape the forms of structural and interpersonal violence they often endure. One example identified the dependence of immigrant women on their spouse for legal status, which, along with structural language and cultural barriers to seeking help, created overlapping patterns of subordination that make immigrant women particularly vulnerable to domestic violence [17].

In the last decade, there has been considerable growth in the application of intersectionality to quantitative research [15], allowing for the examination of the effects of multiple, intersecting dimensions of social identity/position and social processes on health outcomes [19–23], perception of health status [23–26], health-related behaviours [27–31], and utilization of health care services [32–35]. Some have demonstrated the presence of statistical interactions labelled “intersectional effects”, whether synergistic (i.e., combined effect is greater than the sum of independent effects) or antagonistic (combined effect is smaller than the sum of independent effects) [21, 25–27, 30, 34, 36]; others have shown a lack of effect beyond additive main effects [23, 37]. Despite the uptake of intersectionality in Canadian population health research, most of the work has focused on health outcomes with few studies examining measures of health care access [31]. Identifying differences in and determinants of health care access can create opportunities for intervention, for example, more equitable public health and policy recommendations. Therefore, this research addressed this gap by applying an intersectionality framework both in conceptualization and data analysis, and using a statistical technique, multilevel analysis of individual heterogeneity and discriminatory accuracy (MAIHDA), to investigate social identities/positions associated with access to primary care.

The objectives of this study were: (1) To determine the extent to which intersections (cross-classified dimensions of gender, age, immigration status, race, and income) can be used to classify those who had a primary care provider and those who did not; (2) To determine the degree to which each dimension contributes to the variance in the likelihood of having a primary care provider between intersections; and (3) To report the predicted probability of having a primary care provider by intersection.

## Methods

### Study design and population

Data were obtained from the 2015–2019 cycles of the Canadian Community Health Survey (CCHS), an annual cross-sectional survey that collects health-related data on individuals 12 years of age or older living in Canada [38]. The CCHS sampling frame covers up to 97–98% of this population and excludes those living on reserves and other Indigenous settlements, full-time Canadian Forces members, institutionalized individuals, children in foster care, and individuals living in some remote regions [38]. The five CCHS cycles were combined using the pooled approach [39]. Each cycle contained its own sampling weights. Sampling weights were standardized by dividing each respondent’s survey weight within each cycle by the mean of that cycle.

CCHS respondents and respondents by proxy were included in this study if they were 18 years of age or older. Those residing in Canadian territories were excluded because the income distribution variable was not available. Small sample sizes of Indigenous populations in the provinces, and their non-representativeness, led to the exclusion of individuals who identified as First Nations, Métis, or Inuk (Inuit).

### Variables

The outcome for this study was having a regular primary care provider, which represents a measure of potential access to primary care [40].

The independent variables used in this study (mapped to Andersen and Newman’s framework) represented five social identity/positions: gender (predisposing), age (predisposing), immigration status (enabling), race (predisposing), and income (enabling). These variables were used to construct the intersections required in MAIHDA analysis and were also included as main effects in the regression models. These variables, called dimensions in intersectional analyses [20, 41, 42], were analyzed and interpreted not as inherent risk factors, but as proxies for experiences arising from power relations and structural processes (e.g., racism, sexism, ageism) that are associated with these social identities/positions [43]. The MAIHDA statistical technique requires that the dimensions are categorized rather than continuous [44]. Each stratum represented one unique intersection along the dimensions of gender, age, immigration status, race, and income (e.g., woman + young adult + non-immigrant + Black + middle income). Based on intersections of gender (2 groups), age (3 groups), immigration status (3 groups), race (6 groups), and income (3 groups), 324 theoretical intersections (2 x 3 x 3 x 6 x 3) were constructed.

In the CCHS, sex was restricted to two responses, male and female. In the absence of gender data, sex was used to represent gender. For this study, age was classified into three balanced and socially meaningful groups (young adult: 18–39 years, middle-aged adult: 40–59 years, and older adult: 60+ years) [41]. Immigration status was classified into three conventionally acknowledged groups (recent immigrant: 0–9 years, established immigrant: 10–121 years, and non-immigrant). Six racial groups were formed based on how race is understood and used as a social descriptor (i.e., categories not based in biology, but constructed by society) in Ontario: South Asian, Arab/West Asian, Chinese/Filipino/Southeast Asian/Korean/Japanese, Latin American, Black, and White [45]. Those who selected *Other* were excluded and those who selected more than one racial/cultural group were classified into the largest (highest frequency in the dataset) racial/cultural group they had selected [46]. Regarding income, individuals were classified into three groups based on household income decile (low income: lowest 30%, middle income: middle 40%, and high income: top 30%).

### Statistical analyses

R 3.6.1 was used to conduct the analyses [47]. Standardized sampling weights were applied in the univariate, bivariate, and multilevel analyses. One thousand bootstrap replicates provided by Statistics Canada were used to compute estimates of precision in the multilevel analysis [38]. All released and reported values were obtained by applying standardized sampling weights to each respondent.

Univariate analysis for each variable and bivariate analyses between each dimension and the outcome variable were conducted. Absolute standardized differences for dichotomous variables, typically used to compare baseline balance between subjects in treatment/exposure groups [48], were calculated for each variable. The standardized difference, compared to the p-value, is less influenced by the sample size and conveys information about effect size [48].

The MAIHDA analysis followed the approach adapted for binary outcomes (multilevel logistic regression), that was employed by previous researchers [20, 41, 42]. However, instead of the Bayesian methods used in these studies, we used frequentist methods. Previous research has demonstrated that the level of bias in estimates was low and comparable between frequentist and Bayesian approaches to multilevel modelling [49]. Level 2 units in the multilevel analysis were the intersections and level 1 units were the individual respondents.

Of note, a stimulation study by Lizotte et al. cautioned against uncritical interpretation of what are sometimes termed “intersectional effects” (i.e., advantage or disadvantage), captured by stratum random effects from MAIHDA methods. This simulation study concluded that fixed effects in MAIHDA reflect “population average effects under an implicit re-weighting of the data giving all intersections are of equal size”, thus the stratum random effects, which utilize these fixed effects as a reference point may not have a clear and relevant interpretation regarding intersectional advantage or disadvantage. Therefore, in contrast to previous studies that have employed MAIHDA [29, 41, 42], we focused on the intersectional predicted probability instead of the difference in probabilities between additive and intersectional methods. Simulation studies have shown MAIHDA to perform well in predicting intersection-specific binary outcomes [50].

#### Objective 1: Discriminatory accuracy

An unadjusted, random intercept model was fitted, where individuals (level 1) were clustered within intersections (level 2) (**Model 1**). There were 324 predetermined intersections. The intra-class correlation coefficient (ICC) was calculated to obtain a measure of discriminatory accuracy, i.e., the ability to distinguish those who had a primary care provider from those who did not, based only on their intersection. A higher ICC indicates greater discriminatory accuracy. The following criteria were considered for ICC (%): no discriminatory accuracy (0–1%), poor discriminatory accuracy (> 1 to ≤ 5%), fair discriminatory accuracy (> 5 to ≤ 10%), good discriminatory accuracy (> 10 to ≤ 20%), very good discriminatory accuracy (> 20 to ≤ 30%), and excellent discriminatory accuracy (> 30%) [51]. To calculate ICC, let $\sigma_{u}^{2}$, represent the between-intersection variance of Model 1 and let $\frac{\pi^{2}}{3}$ represent the variance of a standard logistic distribution [52].


$$ICC=\frac{\sigma_{u}^{2}}{\sigma_{u}^{2}+\left(\frac{\pi^{2}}{3}\right)}$$

#### Objective 2: Individual intersectional dimension contributions to variance

Five partially adjusted multilevel models (**Models 2a-2e**) were constructed to explore the extent to which each dimension (i.e., gender, age, immigration status, race, and income) in the intersections contributed to the between-intersection variance in **Model 1**; in other words, which dimensions were most and least important in the ability to distinguish those who had a primary care provider from those who did not, based on the intersections [20]. This was done by including each independent variable individually as a main (fixed) effect in separate models (resulting in 5 models, **Models 2a through 2e**) and including the random intercept for the intersections as was done in **Model 1**. The extent to which each dimension contributed to the between-intersection variance was measured first by calculating the ICC for each of **Models 2a through 2e**. This indicated the resulting residual between-intersection variance (of the between-intersection variance determined in **Model 1**), after having adjusted for each main effect. Additionally, the proportional change in the between-intersection variance (PCVs) between each new model and **Model 1** was calculated. To calculate PCV, let $\sigma_{uj(1)}^{2}$represent the variance of the intersections of **Model 1** and let $\sigma_{uj(2)}^{2}$ represent the variance of the intersections with main effects of each **Model 2a through 2e**.


$$PCV=\frac{\sigma_{uj(1)}^{2}-\sigma_{uj(2)}^{2}}{\sigma_{uj(1)}^{2}}$$

The PCV indicates the proportion of the between-intersection variance that was determined in Model 1 that is now explained by having added the main effect. A low PCV suggests that there is very little reduction in between-intersection variance in moving from Model 1 to Model 2a through 2e.

#### Objective 3: Intersectional predicted probability

To build the full multilevel model, **Model 3,** all main effects of the five independent variables (i.e., gender, age, immigration status, race, and income) were added to Model 1 as fixed effects. Odds ratios and 95% confidence intervals were computed.

The ICC was computed to capture the remaining between-stratum variance (random effect) following the adjustment for the main effects. A lower ICC indicates a higher explanatory power held by the simple adjustment of main effects. The PCV was also computed which indicates the proportion of the between-intersection variance (previously determined in **Model 1)** that is now explained by having added all five main effects (**Model 3**), i.e., the reduction in between-intersection variance resulting from moving from **Model 1** to **Model 3**.

Intersectional predicted probabilities (based on fixed and random effects of **Model 3**) for each stratum were calculated to represent outcome predictions of whether one had or did not have a primary care provider for each stratum. To compute intersectional predicted probabilities, the stratum-specific log odds from both the additive and the random effects of **Model 3** were obtained and transformed into predicted probabilities.

## Results

### Descriptive analyses

Of the pooled population, 11.6% was removed according to exclusion criteria (<18 years old, residing in a territory, or Indigenous respondent), and an additional 3.5% was removed during listwise deletion. A total of 241,445 individuals were included in the final analytic sample.

**Table 1** reports the weighted descriptive statistics for the five independent variables. Overall, 84.7% of individuals had a primary care provider. Cell sizes for each stratum could not be released from the RDC due to small numbers; descriptive statistics for these are not provided. Small cell sizes occurred most frequently in strata with non-immigrant or recently immigrated older adults who were Latin American, South Asian, or West Asian. Further, four intersections did not meet the minimum sample size requirements (an unweighted frequency of 5) to be included in the analysis. Thus, only 320 intersections were analyzed.

**Table 1:** Weighted study characteristics.

	Total	Did not have PC provider	Had PC provider	Absolute
				Standardized
	n = 241445	n = 37035 (15.3%)	n = 204410 (84.7%)	**Difference (%)**
	n (%)	n (%)	n (%)	
**Gender**				29.0
Man	118764 (49.2)	22695 (61.3)	96070 (47.0)	
Woman	122681 (50.8)	14340 (38.7)	108340 (53.0)	
**Age**				
Young adult (18–39 years)	87640 (36.3)	21593 (58.3)	66047 (32.3)	54.1
Middle-aged adult (40–59 years)	82852 (34.3)	10701 (28.9)	72150 (35.3)	13.7
Older adult (60+ years)	70953 (29.4)	4740 (12.8)	66213 (32.4)	48.2
**Immigration status**				
Non-immigrant	182287 (75.5)	27880 (75.3)	154407 (75.5)	0.5
Recent immigrant	15337 (6.35)	4240 (11.4)	11097 (5.4)	21.8
Established immigrant	43820 (18.1)	4915 (13.3)	38906 (19.0)	15.5
**Race**				
Black	6924 (2.9)	1808 (4.9)	5116 (2.5)	12.7
East Asian	20803 (8.6)	3820 (10.3)	16983 (8.3)	6.9
Latin American	3536 (1.5)	812 (2.2)	2724 (1.3)	6.9
South Asian	12188 (5.1)	1863 (5.0)	10325 (5.1)	0.5
West Asian	5013 (2.1)	1293 (3.5)	3719 (1.8)	10.6
White	192982 (79.9)	27438 (74.1)	165544 (81.0)	16.6
**Income**				
Low	68578 (28.4)	13593 (36.7)	54984 (26.9)	21.2
Middle	97445 (40.4)	14264 (38.5)	83181 (40.7)	4.5
High	75422 (31.2)	9177 (24.8)	66245 (32.4)	16.9

PC: Primary care. All values are n (%). Chi-square tests of independence were conducted for each variable; all p-values were <0.01. Absolute standardized differences were calculated and expressed as percentages. Immigration status categories were defined as recent immigrant (0–9 years) and established immigrant (10–121 years). Income categories were defined as low (bottom 30%), middle (middle 40%), high (upper 30%).

Bivariate analyses using chi-square tests of independence to assess the association between the outcome and independent variables are reported in **Table 1**. A standardized difference of 29% indicates a small effect and approximately 20% of non-overlap between men and women, with women being more likely than men to have a primary care provider [53]. Those who did not have a primary care provider were generally younger in age. Recent immigrants were overrepresented among those who did not have a primary care provider, when compared against their relative proportions in the study sample; the opposite was true for established immigrants. All visible minority groups, except for South Asian individuals, were overrepresented among those who did not have a primary care provider, when compared to their relative proportion in the sample. Finally, those who had lower income were overrepresented among those who did not have a primary care provider.

### Multilevel Analysis of Individual Heterogeneity and Discriminatory Accuracy (MAIHDA)

#### Objective 1: Discriminatory accuracy

The intra-class correlation coefficient (ICC) for the unadjusted, random intercept model was 23.2% (95% CI: 20.5, 26.1%), indicating very good discriminatory accuracy. In other words, 23.2% of the total individual differences in whether someone had or did not have a primary care provider was attributed to the intersections to which individuals belonged.

#### Objective 2: Individual intersectional dimension contributions to variance

The contribution of dimensions to between-intersection variance (**Model 1 vs Model 2a-2e**) in order from largest to smallest were: age (PCV: 48.4%; 95% CI: 42.0, 54.9%), immigration status (PCV: 17.8%; 95% CI: 17.3, 18.2), income (PCV: 5.9%; 95% CI: 4.0, 8.5), race (PCV: 5.1%; 95% CI: 3.9, 6.7), and gender (PCV: 5.0%; 95% CI: 4.0, 6.2). This means, as an example, only 5% of between-intersection variance in whether one had a primary care provider was explained by gender.

#### Objective 3: Predicted probability based on intersectional methods

Adjusting for all five independent variables reduced the ICC for **Model 3** to 5.9% [95% CI: 2.0, 16.1]) from the null **Model 1** (ICC: 23.2%; 95% CI: 20.5, 26.1%). This means there was 5.9% of residual individual differences in having/not having a primary care provider that were related to intersections, and that were unexplained by adjusting for the main effects of gender, age, immigration status, race, and income. Both the ICC and the PCV for **Model 3** provides evidence that the additive model did not adequately capture all of the variability between intersections.

**Table 2** reports the odds ratios for the fixed effects in **Model 3** and the ICC following adjustment. Intersections containing women compared with intersections containing men (OR: 1.52; 95% CI: 1.35, 1.72); Intersections containing middle-aged (OR: 2.29; 95% CI: 1.93, 2.72) and intersections containing older adults (OR: 2.29; 95% CI: 1.93, 2.72) compared with intersections containing young adults; intersections containing established immigrants compared with intersections containing non-immigrants (OR: 1.60; 95% CI: 1.37, 1.86); intersections containing East Asian (OR: 1.60; 95% CI: 1.37, 1.86) and intersections containing South Asian (OR: 1.82; 95% CI: 1.46, 2.26) compared with intersections containing Black individuals; and intersections containing middle income (OR: 1.46; 95% CI: 1.26, 1.68) and intersections containing high income (OR: 1.78; 95% CI: 1.53, 2.08) compared with intersections containing low income individuals were more likely to have a primary care provider.

**Table 2:** Model 3 summary.

		Model 3
		OR (95% CI)
**Gender**	Male	Ref.
	Female	1.52 (1.35, 1.72)**
**Age**	Young	Ref.
	Middle	2.29 (1.93, 2.72)**
	Old	4.80 (4.05, 5.70)**
**Immigration**	Non-immigrant	Ref.
**status**	Recent immigrant	0.81 (0.69, 0.95)*
	Established immigrant	1.60 (1.37, 1.86)**
**Race**	Black	Ref.
	East Asian	1.29 (1.05,1.59)*
	Latin American	1.16 (0.92, 1.47)
	South Asian	1.82 (1.46, 2.26)**
	West Asian	1.06 (0.84, 1.33)
	White	1.18 (0.97, 1.45)
**Income**	Low	Ref.
	Middle	1.46 (1.26, 1.68)**
	High	1.78 (1.53, 2.08)**
**ICC (95% CI)**		5.9 (2.0, 16.1)

*P<0.05

**P<0.001 OR: odds ratio CI: confidence ICC: intra-class correlation coefficient

**Model 3** is a fully adjusted, random intercept model, wherein the level-1 units were the individual respondents, and the level-2 units were intersections. Main effect estimates are expressed in odds ratios and 95% confidence intervals. Age categories were defined as young adult (18–39 years), middle-aged adult (40–59 years), and older adult (60+ years). Immigration status categories were defined as recent immigrant (0–9 years) and established immigrant (10–121 years). Income categories were defined as low (bottom 30%), middle (middle 40%), high (upper 30%).

**Fig 1:**
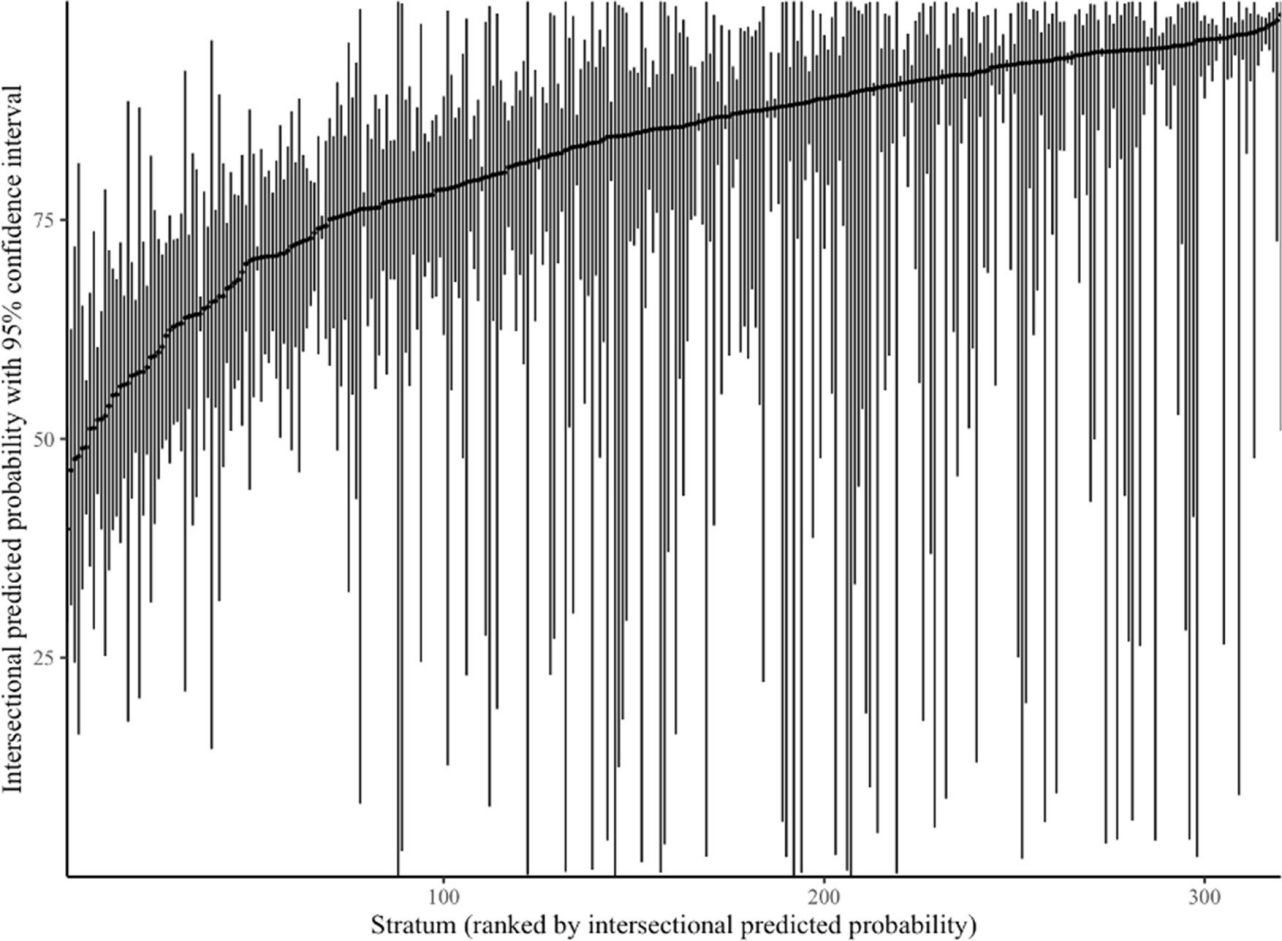
Stratum-specific intersectional predicted probability (and 95% confidence interval) of having a PC provider. PC: Primary care. Intersectional predicted probabilities are based on the **Model 3**, a multilevel regression analysis wherein the level-1 units were the individual respondents, and the level-2 units were intersections. This model was fully adjusted for the individual dimensions used to construct the intersections (i.e., gender, age, immigration status, race, income). Age categories were defined as young adult (18–39 years), middle-aged adult (40–59 years), and older adult (60+ years). Immigration status categories were defined as recent immigrant (0–9 years) and established immigrant (10–121 years). Income categories were defined as low (bottom 30%), middle (middle 40%), high (upper 30%). Intersections are ranked by the size of intersectional predicted probabilities. Corresponding intersection descriptions can be found in **S1A Table**.

**Table 3** reports the ten lowest and ten highest stratum-specific intersectional predicted probabilities of having a primary care provider along with stratum descriptions that correspond to each ranking. **Fig 1** graphs the intersectional predicted probabilities for the 320 intersections, ranked from the stratum with the lowest intersectional predicted probability to the highest. **S1A Table** reports the stratum descriptions that correspond to each ranking in **Fig 1** along with the intersectional predicted probabilities for each stratum. Intersectional predicted probabilities ranged from 39.70% to 98.44%. As an example, the lowest intersectional predicted probability of having a primary care provider was for recently immigrated young adult Black men with low income; whereas, the highest intersectional predicted probability for having a primary care provider was for established immigrant, older South Asian women with high income. The width of predicted probability confidence intervals varied greatly across intersections, ranging from CI: 89.33, 94.89% in established immigrant, middle-aged White women with high income to CI: 0, 100.00% in non-immigrant, older Latin American women with middle income. The widest 10% and narrowest 10% of confidence intervals with corresponding intersections can be found in **S1B and S1C Table**, respectively.

**Table 3:** Lowest and highest intersection-specific intersectional predicted probabilities (and 95% CIs) of having a PC provider.

						Predicted probability Intersectional (%)	
					
Ranking	Gender	Age	Immigration status	Race	Income	Point estimate	95% CI
1	Man	Young adult	Recent immigrant	Black	Low	39.70	29.08, 51.37
2	Man	Young adult	Non-immigrant	Latin American	Low	46.40	30.97, 62.56
3	Man	Young adult	Recent immigrant	West Asian	High	47.69	24.42, 72.00
4	Man	Young adult	Non-immigrant	West Asian	High	48.00	16.2, 81.50
5	Man	Young adult	Non-immigrant	West Asian	Low	48.92	32.83, 65.23
6	Woman	Young adult	Non-immigrant	East Asian	Low	49.04	41.4, 56.73
7	Man	Young adult	Recent immigrant	Black	Middle	51.16	35.39, 66.70
8	Man	Young adult	Recent immigrant	Black	High	51.24	28.24, 73.72
9	Man	Young adult	Non-immigrant	East Asian	Low	52.16	43.7, 60.50
10	Man	Young adult	Non-immigrant	South Asian	Low	52.26	39.64, 64.59
310	Man	Older adult	Established immigrant	East Asian	Middle	96.18	93.31, 97.85
311	Man	Older adult	Established immigrant	South Asian	High	96.21	82.53, 99.27
312	Woman	Middle-aged adult	Established immigrant	South Asian	Low	96.36	90.86, 98.60
313	Woman	Older adult	Established immigrant	Latin American	Middle	96.41	47.76, 99.87
314	Man	Older adult	Established immigrant	East Asian	High	96.63	92.61, 98.49
315	Woman	Older adult	Established immigrant	West Asian	High	96.77	94.24, 98.21
316	Woman	Older adult	Established immigrant	Black	High	96.91	95.05, 98.09
317	Woman	Older adult	Established immigrant	Latin American	High	97.27	94.38, 98.69
318	Woman	Older adult	Established immigrant	East Asian	High	97.45	91.86, 99.23
319	Woman	Older adult	Established immigrant	South Asian	Low	97.79	72.57, 99.86
320	Woman	Older adult	Established immigrant	South Asian	High	98.44	50.92, 99.97

PC: Primary care. Intersectional predicted probabilities were obtained from **Model 3**, a multilevel regression analysis wherein the level-1 units were the individual respondents, and the level-2 units were intersections. This model was fully adjusted for the individual dimensions used to construct the intersections (i.e., gender, age, immigration status, race, income). Age categories were defined as young adult (18–39 years), middle-aged adult (40–59 years), and older adult (60+ years). Immigration status categories were defined as recent immigrant (0–9 years) and established immigrant (10–121 years). Income categories were defined as low (bottom 30%), middle (middle 40%), high (upper 30%). Strata are ranked by the size of predicted probabilities. Only the ten lowest and ten highest ranked intersections are displayed; a full table can be found in **S1A Table**.

## Discussion

This study advances social epidemiology as it was guided by an intersectionality framework and utilized a compatible statistical technique, MAIHDA, instead of the conventional approach used in health research of considering dimensions only as independent and additive. This study is among the few Canadian studies that applied intersectionality using the MAIHDA approach to study health-related outcomes [25, 54].

### Key findings

In this study, the discriminatory accuracy was found to be 23%, which provides evidence that intersections explained a substantial amount of the individual variation in whether one had a primary care provider and raises concerns regarding inequity. The relative contributions of each dimension to the between-intersection variance, in order from largest to smallest contribution, were age, immigration status, income, race, and gender. Finally, the intersection-specific predicted probabilities (frequencies) of having a primary care provider were determined and are discussed further below. The ICC in Model 3 of 5.9% is lower than that found in the null model of 23%; however, it is statistically significantly greater than zero, indicating the presence of between-intersection variability in whether one had a primary care provider that was not entirely accounted for by the main effects. Thus, access to primary care not only varies greatly among individuals of different social identities/positions, but they vary at different intersections of these identities/positions.

Regarding main effects from **Model 3**, the odds of having a primary care provider were higher for: women compared with men, older and middle-aged adults compared with young adults, established immigrants compared with non-immigrants, South Asian individuals compared with Black individuals, and those with high income compared with those with low income. Non-intersectional studies that investigated sociodemographic determinants of having a primary care provider by analyzing these variables using either unitary (i.e., focus on single dimension) or multiple (i.e., analyze multiple dimensions as independent and additive) approaches have generally been concordant with our results. In a study by Talbot et al., women were more likely to have a regular doctor, compared with men [10]. Older respondents were more likely than younger respondents to have a regular doctor [10]. Regarding immigration status, the findings were mixed. Inconsistent with our study, two studies found that those who were native born (in Canada) were more likely than foreign born to have a primary care provider [8, 9]. However, in other studies wherein the “foreign-born” category was deconstructed into recent (less than ten years in Canada) and established immigrants (ten or more years in Canada), recent immigrants were less likely to have a regular medical doctor than non-immigrants and established immigrants, while established immigrants were more likely than non-immigrants to have a regular doctor [10, 55]. Regarding race, no study could be directly compared to ours due to differences in the granularity of the non-White race categories; one study found no differences between White and non-White individuals (when racial minorities were all grouped into a non-White category) with respect to having a primary care provider [8]. Similar to our findings, there is evidence that those in the highest income quintile are more likely to have a primary care provider than individuals in the lowest income quintile [8, 10]. A study by Lebrun and Shi, however, did not find evidence of an association between income quintile and having a regular medical doctor [9].

To further interpret **Model 3** considering both fixed and random effects, the intersectional predicted probability of having a primary care provider for each intersection was obtained. The highest intersectional predicted probability of having a primary care provider belonged to established immigrant, older South Asian women with high income. The lowest intersectional predicted probability of having a primary care provider was for recently immigrated, young, Black men with low income.

### Examination of the lowest predicted probability using an intersectionality framework

It is instructive to examine this lowest predicted probability through an intersectionality lens. For recently immigrated, young, Black men with low income in Canada, primary care access can be affected by intersecting forces of structural racism and xenophobia, racial discrimination, and having low income, as well as the influence of social norms at the intersection of gender, age, and race.

Structural racism refers to the interaction of macrolevel systems, social forces, institutions, ideologies, and processes that results in racialized outcomes [56]. Structural racism is seen in the social segregation that new Black immigrants to Canada face. According to data collected in 2016, nearly 95% of Black individuals lived in Canada’s Census Metropolitan Areas, compared with 71 of the overall population [57]. Within these Census Metropolitan Areas, new immigrants (or visible minorities) were more likely to settle in new or peripheral neighbourhoods with more affordable housing [58]. A study investigating geographic accessibility to primary health care services in these kinds of Canadian urban settings found that neighbourhoods with poor accessibility scores often overlapped with neighbourhoods in which a high concentration of recent immigrant and other socially disadvantaged groups (e.g., lone-parent, low education) resided [58]. These quantitative results reflected the sentiments of participants in qualitative studies [59, 60]. In one study of access to primary care services for African immigrant families living in Manitoba, transportation was a major challenge as many did not have cars; it was noted that “[f]amilies could not reach out for care because they were new to the city and relied on public buses that only operated on certain schedules and on specific routes” [60]. In a study focused on a Toronto neighbourhood with a large proportion of new immigrants, participants also identified geographic access to family physicians as a barrier; instead participants opted for walk-in clinics and hospitals for primary care [59]. During the cold winter months, low-income families experienced the added barrier of acquiring winter clothing for the travel [60].

Structural xenophobia (i,e., the exclusionary attitudes and behaviours towards those who are perceived to have a cultural or national identity that is foreign to the host country [61]) manifests in the Canada Health Act, which has imposed a 3-month wait period on a newcomer (e.g., resident moving from one province/territory to another, returning Canadian, permanent resident, foreign worker) to various provinces (Ontario, British Columbia, Manitoba, New Brunswick, Quebec) before they can be covered by provincial health plans [62]. During this period, immigrants are left either to purchase private insurance, pay out of pocket, or forego seeking medical care [59]. There is a paucity of research regarding the effects of the 3-month wait period on the long-term health care access and health outcomes of newly immigrated Black men to Canada. However, a qualitative study evaluating its impact on immigrant women to British Columbia has uncovered themes of feeling stigmatized, dehumanized, and powerlessness, which resulted in an early mistrust in the health care system and unmet health care needs [63]. A scoping review examining the outcomes of the 3-month wait in Ontario has also supported the theory that a limited access to care early in an immigrant’s journey in Canada may have implications on their trust, ability to navigate, and a delay in accessing the health care system [62].

Racial discrimination, on the macro- and micro-level has been elucidated as a recurring barrier for visible minorities, particularly new immigrants, in accessing health care. Most commonly and evidently, language barriers created a source of tension, especially when patients felt that health care providers were not interested in helping them understand health care procedures or could not adequately provide translation services to convey the diagnosis and treatment plan [60, 64, 65]. More insidiously and specific to African, Caribbean, and Black immigrant populations, distrust in the health care system is oftentimes rooted in a history of colonization of African, Caribbean, and Black individuals’ countries of origin [66]. This distrust is sustained in a Black immigrant’s journey through the Canadian health care system in several ways as described by Fante-Coleman et al., 2022; [Many patients felt that] “physicians lacked knowledge about their culture, histories, and needs”, and thus, could not attend to their health care needs. Furthermore, physicians lacked an awareness of the social causes (e.g., racism and generational trauma) underlying some patients’ illness. More broadly, “the inability to relate to patients and their social world led to frustration and alienated respondents from the healthcare system [66].”

Gendered attitudes and behaviours towards health is a phenomenon that has been theorized and studied to explain consistently poorer health outcomes seen in men [67]. Across the lifespan, masculinity typically manifests differently [67]. In young men, physical risk, including risk-taking behaviours is naturalized and celebrated. Paired with the propensity to monitor their own symptoms instead of seeking help, health-seeking behaviours are often minimized. Despite this pattern among all men generally, there have been observed variations in the embodiment of masculinity among ethnic and racial groups [68]. A US study looked at the mediating role of self-stigma in the association between conformity to dominant US masculine norms and attitudes towards counseling across cultures. Gendered contexts were important contributors to attitudinal disparities toward help-seeking in African American men, although the pathway by which this occurred was not through self-stigma as the authors hypothesized, and warrants additional research [68]. It is important to note that despite cultural similarities between Canada and the US, there is some evidence of lower adherence to traditional masculine norms in Canadian men, possibly explained by political and ideological differences in acceptance of non-normative gender and sexuality [69].

Much of the literature discussed in this section has explored the experiences of group members at the intersection of certain dimensions; for instance, new Black immigrants (who may have low income) [60, 66], young males [67], Black males [68] or single dimensions like immigration status [59]. However, taken together, the interlocking of social forces that have contributed to these various experiences provides support for our finding that recently immigrated, young, Black men with low income had the lowest predicted probability of having a primary care provider.

### Contributions and future directions for health research

The introduction of intersectional frameworks into quantitative research methods using techniques like MAIHDA can inform the further development of conceptual frameworks relating to health service utilization. In Andersen and Newman’s discussion of the Framework of Health Services Utilization, the “need to consider the possibility of interaction effects between two or more variables in planning for social change” was noted [12]. Due to its consideration of multiple factors in Andersen and Newman’s framework, this study can provide a more complete mapping of the relationships between the predisposing and enabling factors, which are often conceptualized as independent entities.

A challenge of this study was understanding whether someone did not have a primary care provider because they truly did not perceive a need or whether macro-level elements (e.g., socio-cultural environments, including policies and institutions) presented barriers to having a primary care provider. Controlling for the effects of a subjective or an objective measure of need may explain away the real and meaningful ways in which social forces (e.g., cultural norms, class discrimination) affect attitudes and outcomes surrounding health. On the other hand, completely attributing inequalities in having a primary care provider across intersections to macro-level elements may ignore the role of personal agency. For instance, if lack of need for a primary care provider leads to personal preferences due to informed decisions or of good health owing to healthy behaviors, not having a primary care provider may not reflect inequity. Future avenues for health services research could look at the mediating role of perceived health/need and personal preferences in the relationship between social identities/positions and access to primary care. Additionally, conducting follow-up qualitative studies, wherein intersections that we have identified as vulnerable, could provide context, descriptive illustrations, and a diversity of views to enrich the interpretation of quantitative results.

### Contributions to praxis

Core to intersectionality is its praxis [70]. In the context of public health, this can be defined as “the practical application of intersectionality to facilitate equitable health policy and practice for intersectionally marginalized groups” [71]. This study identified strata with the lowest intersectional predicted probabilities of having a primary care provider (e.g., recently immigrated young Black men with low income). From a health policy and promotion perspective, continued efforts should be made towards more equitable economic and social distribution of health care resources for individuals within strata with a low, intersectional predicted probability of having a primary care provider. Addressing more specifically the lack of access to care, building community partnerships in communities where individuals are at risk of not having a primary care provider could foster co-created initiatives to draw on the strengths, knowledge, and resources of communities. These collaborations can help to create culturally relevant programs and services for African immigrant and refugee populations [60]. As an example, the Toronto-based community health centre, TAIBU, offers “Black-identifying clients from throughout the Greater Toronto Area access to primary care, health promotion and disease prevention programs in a culturally affirming environment.” Given the intersectional importance of age as a dimension, TAIBU provides community programming across the lifespan, including those aimed at supporting youth [72].

Importantly, these partnerships may offer an understanding of the unique needs of marginalized groups to ensure appropriate messaging (instead of messaging that has historically served privileged groups). For example, early HIV risk reduction messaging targeted towards racialized women has been critiqued for its centering of White middle-class experiences [73]. For many populations that were most at risk (particularly poor, urban, Black and Latina women), the suggestions were not realistic, given the cultural norms and context of their lives [73]. Thus, an understanding of the social power relations faced by members of at-risk intersections must be gained to meaningfully and appropriately apply intersectionality research to public health and health system policies and practice.

### Strengths and limitations

A strength of the study was the large, pooled sample size which allowed for the investigation of many specific intersections, constructed using multiple dimensions, and with a fine-grained measure of race. This was enhanced by the low percentage of missing data in the dataset. A key strength of the analysis plan was employing the intersectionality theoretical framework which accounts for crucial context in determining advantaged or disadvantaged social identities/positions.

One limitation in this study is that within quantitative intersectionality research that relies on categorizing dimensions, there is an expected trade-off between studying a large number of intersections and gaining a deeper understanding of what is occurring within each intersection [74]. Small sample sizes within strata can lead to wider confidence intervals for predicted probabilities. In particular, groups for which confidence intervals were widest were generally found in non-immigrant and recent immigrants of West Asian, Latin American, South Asian, and Black descent, whereas the narrowest confidence intervals were found in white non-immigrants or established immigrants. RDC confidentiality policies precluded the reporting of intersection sizes (particularly for small intersections). As a result, we were unable to disentangle the effects of sample size from within-stratum variability on the size of the confidence interval. This raises a methodological and ethical concern that intersections representing more marginalized groups also have less precise estimates. Another limitation of this study was its pooling across provinces. This precludes examination of inter-province heterogeneity, which can be considerable in Canada with respect to such things as the proportion of individuals that have a primary care provider [75], the degree to which inter-professional team-based approaches are adopted, and provider remuneration structures.

Finally, our results may not generalize to groups who were not surveyed (e.g., individuals living on reserves, in remote areas, and institutionalized individuals), or those who were excluded in this study due to sample size or variable restrictions (e.g., Indigenous respondents, those living in territories). Access to care for these populations may be worse than in the included population, and many of these populations (e.g., Indigenous populations) suffer worse health outcomes as a result of historical and ongoing oppression, colonization, and marginalization within Canada [76]; future research with a sufficient and fully representative sample should be conducted using appropriate methodologies for these populations.

## Conclusion

Drawing from intersectionality theory and Andersen and Newman’s Framework of Health Services Utilization, this study utilized data from the 2015–2019 cycles of the Canadian Community Health Survey and an innovative application of multilevel modelling (MAIHDA) to examine the association between an individual’s location at the intersection of various social identities/positions (based on dimensions of gender, age, immigration status, race, and income) and whether they had a primary care provider. This study provided evidence for the importance of intersections (particularly the dimension of age) in determining individual variation in whether one had a primary care provider. The lowest predicted probability of having a primary care provider was found for recently immigrated, young, Black men with low income. These findings support the need for future research that (1) utilizes quantitative intersectionality methods, (2) improves statistical and theoretical features of current methods, and (3) explores factors driving low predicted probabilities and ultimately informs large scale, targeted initiatives in improving access to primary care.

## Supporting information

S1 Table Intersectional predicted probabilities and corresponding confidence intervals in S1A-S1C Tables were obtained from Model 3, a multilevel regression analysis wherein the level-1 units were the individual respondents, and the level-2 units were intersections. This model was fully adjusted for the individual dimensions used to construct the intersections (i.e., gender, age, immigration status, race, income). Age categories were defined as young adult (18–39 years), middle-aged adult (40–59 years), and older adult (60+ years). Immigration status categories were defined as recent immigrant (0–9 years) and established immigrant (10–121 years). Income categories were defined as low (bottom 30%), middle (middle 40%), high (upper 30%). S1A Table provides a complete list of intersection descriptions, ranked from lowest to highest predicted probability of having a primary care provider. S1B and S1C Tables provide a list of intersections with predicted probabilities that fall within the widest and narrowest 10% of confidence intervals, respectively.
(DOCX)
