# Supplementary material for: Understanding intersectional inequality in access to primary care providers using multilevel analysis of individual heterogeneity and discriminatory accuracy
Source: PLoS One. 2024 Jan 19;19(1):e0296657. doi: 10.1371/journal.pone.0296657 (PMC10798491; doi:10.1371/journal.pone.0296657)
Supplement: S1 Table — This model was fully adjusted for the individual dimensions used to construct the intersections (i.e., gender, age, immigration status, race, income). Age categories were defined as young adult (18–39 years), middle-aged adult (40–59 years), and older adult (60+ years). Immigration status categories were defined as recent immigrant (0–9 years) and established immigrant (10–121 years). Income categories were defined as low (bottom 30%), middle (middle 40%), high (upper 30%). S1A Table provides a complete list of intersection descriptions, ranked from lowest to highest predicted probability of having a primary care provider. S1B and S1C Tables provide a list of intersections with predicted probabilities that fall within the widest and narrowest 10% of confidence intervals, respectively. (DOCX) [file pone.0296657.s001.docx]

# Supporting Tables (S1)

Table A. Ranked intersectional predicted probabilities (and 95% CIs) of having a PC provider

|  |  |  |  |  |  | Predicted probability Intersectional (%) | | |
| --- | --- | --- | --- | --- | --- | --- | --- | --- |
|  |  |  |  |  |  |  |  |  |
| Ranking | Gender | Age | Immigration status | Race | Income | Point estimate | 95% CI |  |
| 1 | Man | Young adult | Recent immigrant | Black | Low | 39.70 | 29.08, 51.37 |  |
| 2 | Man | Young adult | Non-immigrant | Latin American | Low | 46.40 | 30.97, 62.56 |  |
| 3 | Man | Young adult | Recent immigrant | West Asian | High | 47.69 | 24.42, 72 |  |
| 4 | Man | Young adult | Non-immigrant | West Asian | High | 48.00 | 16.2, 81.5 |  |
| 5 | Man | Young adult | Non-immigrant | West Asian | Low | 48.92 | 32.83, 65.23 |  |
| 6 | Woman | Young adult | Non-immigrant | East Asian | Low | 49.04 | 41.4, 56.73 |  |
| 7 | Man | Young adult | Recent immigrant | Black | Middle | 51.16 | 35.39, 66.7 |  |
| 8 | Man | Young adult | Recent immigrant | Black | High | 51.24 | 28.24, 73.72 |  |
| 9 | Man | Young adult | Non-immigrant | East Asian | Low | 52.16 | 43.7, 60.5 |  |
| 10 | Man | Young adult | Non-immigrant | South Asian | Low | 52.26 | 39.64, 64.59 |  |
| 11 | Man | Middle-aged adult | Non-immigrant | Black | Low | 52.62 | 25.22, 78.53 |  |
| 12 | Man | Young adult | Established immigrant | Black | Low | 53.75 | 34.98, 71.52 |  |
| 13 | Woman | Young adult | Recent immigrant | Latin American | Low | 54.97 | 39.58, 69.46 |  |
| 14 | Man | Middle-aged adult | Recent immigrant | White | Low | 55.07 | 41.12, 68.26 |  |
| 15 | Man | Young adult | Non-immigrant | West Asian | Middle | 56.00 | 38.12, 72.44 |  |
| 16 | Man | Young adult | Recent immigrant | White | Low | 56.18 | 45.47, 66.34 |  |
| 17 | Man | Middle-aged adult | Non-immigrant | Latin American | Low | 56.35 | 17.68, 88.59 |  |
| 18 | Man | Young adult | Non-immigrant | Black | Middle | 57.21 | 43.16, 70.18 |  |
| 19 | Woman | Young adult | Recent immigrant | Black | Low | 57.37 | 48.39, 65.88 |  |
| 20 | Woman | Young adult | Recent immigrant | Black | High | 57.63 | 20.34, 87.87 |  |
| 21 | Man | Young adult | Recent immigrant | Latin American | Low | 57.65 | 41.22, 72.54 |  |
| 22 | Man | Young adult | Recent immigrant | White | Middle | 58.16 | 48.2, 67.49 |  |
| 23 | Man | Young adult | Non-immigrant | Black | High | 59.32 | 31.3, 82.36 |  |
| 24 | Woman | Young adult | Non-immigrant | Latin American | Middle | 59.47 | 40.27, 76.15 |  |
| 25 | Man | Young adult | Recent immigrant | West Asian | Middle | 59.88 | 45.39, 72.83 |  |
| 26 | Man | Young adult | Established immigrant | White | Low | 60.50 | 48.85, 71.07 |  |
| 27 | Man | Young adult | Recent immigrant | West Asian | Low | 61.77 | 49.86, 72.42 |  |
| 28 | Man | Middle-aged adult | Recent immigrant | West Asian | Low | 62.43 | 47.18, 75.56 |  |
| 29 | Man | Young adult | Non-immigrant | Black | Low | 62.81 | 51.64, 72.76 |  |
| 30 | Man | Young adult | Recent immigrant | South Asian | Low | 63.05 | 51.96, 72.92 |  |
| 31 | Woman | Young adult | Non-immigrant | West Asian | Low | 63.18 | 48.55, 75.74 |  |
| 32 | Man | Middle-aged adult | Recent immigrant | Black | High | 63.82 | 21.14, 92.07 |  |
| 33 | Woman | Young adult | Recent immigrant | West Asian | Low | 63.96 | 53.42, 73.31 |  |
| 34 | Man | Young adult | Established immigrant | Latin American | Low | 64.10 | 40.09, 82.65 |  |
| 35 | Woman | Young adult | Non-immigrant | Latin American | Low | 64.21 | 43.31, 80.81 |  |
| 36 | Man | Young adult | Non-immigrant | White | Low | 64.29 | 62.27, 66.26 |  |
| 37 | Man | Young adult | Recent immigrant | South Asian | High | 64.89 | 48.69, 78.25 |  |
| 38 | Man | Young adult | Recent immigrant | East Asian | Middle | 65.09 | 54.66, 74.24 |  |
| 39 | Man | Middle-aged adult | Non-immigrant | Black | High | 65.62 | 14.57, 95.53 |  |
| 40 | Man | Middle-aged adult | Recent immigrant | Black | Low | 65.75 | 53.56, 76.17 |  |
| 41 | Man | Young adult | Recent immigrant | Latin American | High | 66.26 | 31.47, 89.36 |  |
| 42 | Man | Young adult | Established immigrant | Black | Middle | 66.29 | 46.75, 81.49 |  |
| 43 | Man | Young adult | Recent immigrant | East Asian | Low | 67.16 | 58.62, 74.69 |  |
| 44 | Woman | Young adult | Non-immigrant | Black | Middle | 67.40 | 50.9, 80.48 |  |
| 45 | Man | Young adult | Recent immigrant | White | High | 67.82 | 55.7, 77.93 |  |
| 46 | Woman | Young adult | Recent immigrant | White | Low | 68.17 | 56.67, 77.82 |  |
| 47 | Man | Young adult | Established immigrant | South Asian | High | 69.04 | 51.49, 82.42 |  |
| 48 | Man | Young adult | Non-immigrant | East Asian | Middle | 69.98 | 62.24, 76.73 |  |
| 49 | Woman | Young adult | Recent immigrant | West Asian | High | 70.32 | 44.17, 87.64 |  |
| 50 | Woman | Young adult | Established immigrant | West Asian | Low | 70.54 | 54.74, 82.58 |  |
| 51 | Man | Young adult | Non-immigrant | White | Middle | 70.62 | 69.21, 71.99 |  |
| 52 | Man | Young adult | Established immigrant | West Asian | Middle | 70.71 | 54.21, 83.12 |  |
| 53 | Man | Young adult | Established immigrant | East Asian | Low | 70.82 | 59.65, 79.95 |  |
| 54 | Woman | Young adult | Non-immigrant | South Asian | Low | 70.85 | 58.62, 80.66 |  |
| 55 | Woman | Young adult | Recent immigrant | White | Middle | 70.85 | 62.3, 78.14 |  |
| 56 | Man | Young adult | Recent immigrant | East Asian | High | 70.89 | 56.88, 81.8 |  |
| 57 | Woman | Middle-aged adult | Non-immigrant | Black | Low | 71.16 | 50.11, 85.84 |  |
| 58 | Woman | Young adult | Non-immigrant | Black | Low | 71.17 | 60.87, 79.67 |  |
| 59 | Woman | Young adult | Recent immigrant | Black | Middle | 71.49 | 55.65, 83.37 |  |
| 60 | Man | Young adult | Recent immigrant | Latin American | Middle | 71.97 | 48.68, 87.43 |  |
| 61 | Woman | Young adult | Established immigrant | Black | Low | 72.22 | 60.44, 81.56 |  |
| 62 | Man | Middle-aged adult | Recent immigrant | Black | Middle | 72.37 | 46.18, 88.89 |  |
| 63 | Man | Young adult | Non-immigrant | South Asian | Middle | 72.59 | 59.95, 82.41 |  |
| 64 | Man | Young adult | Recent immigrant | South Asian | Middle | 72.70 | 62.6, 80.91 |  |
| 65 | Man | Young adult | Established immigrant | White | Middle | 72.92 | 65.18, 79.49 |  |
| 66 | Woman | Young adult | Recent immigrant | East Asian | Low | 73.52 | 66.84, 79.28 |  |
| 67 | Man | Young adult | Non-immigrant | South Asian | High | 74.01 | 59.66, 84.58 |  |
| 68 | Man | Young adult | Non-immigrant | White | High | 74.17 | 72.79, 75.49 |  |
| 69 | Man | Middle-aged adult | Established immigrant | West Asian | Low | 74.33 | 61.41, 84.04 |  |
| 70 | Woman | Young adult | Recent immigrant | West Asian | Middle | 75.07 | 58.34, 86.62 |  |
| 71 | Woman | Young adult | Recent immigrant | East Asian | High | 75.20 | 62.62, 84.58 |  |
| 72 | Man | Young adult | Established immigrant | Latin American | High | 75.30 | 48.67, 90.74 |  |
| 73 | Woman | Young adult | Recent immigrant | South Asian | High | 75.43 | 55.96, 88.13 |  |
| 74 | Man | Middle-aged adult | Recent immigrant | White | Middle | 75.60 | 63.56, 84.63 |  |
| 75 | Man | Middle-aged adult | Recent immigrant | Latin American | Low | 75.69 | 32.52, 95.26 |  |
| 76 | Man | Young adult | Non-immigrant | Latin American | Middle | 75.86 | 55.03, 88.97 |  |
| 77 | Woman | Middle-aged adult | Recent immigrant | Black | Middle | 76.03 | 43.11, 93 |  |
| 78 | Man | Middle-aged adult | Non-immigrant | West Asian | Low | 76.25 | 8.32, 99.13 |  |
| 79 | Man | Middle-aged adult | Non-immigrant | White | Low | 76.28 | 74.25, 78.19 |  |
| 80 | Woman | Middle-aged adult | Recent immigrant | White | Low | 76.29 | 62.87, 85.95 |  |
| 81 | Woman | Young adult | Established immigrant | White | Low | 76.33 | 65.98, 84.28 |  |
| 82 | Man | Middle-aged adult | Non-immigrant | East Asian | Middle | 76.39 | 55.66, 89.29 |  |
| 83 | Man | Young adult | Established immigrant | West Asian | Low | 76.42 | 59.51, 87.73 |  |
| 84 | Man | Young adult | Established immigrant | East Asian | Middle | 76.85 | 69.16, 83.09 |  |
| 85 | Woman | Young adult | Established immigrant | Latin American | Middle | 77.05 | 57.33, 89.35 |  |
| 86 | Man | Young adult | Established immigrant | White | High | 77.11 | 68.2, 84.1 |  |
| 87 | Man | Young adult | Established immigrant | East Asian | High | 77.14 | 68.18, 84.16 |  |
| 88 | Man | Middle-aged adult | Non-immigrant | West Asian | Middle | 77.29 | 0.1, 99.99 |  |
| 89 | Woman | Older adult | Recent immigrant | West Asian | Low | 77.35 | 2.9, 99.74 |  |
| 90 | Woman | Middle-aged adult | Recent immigrant | Black | Low | 77.42 | 59.82, 88.76 |  |
| 91 | Man | Middle-aged adult | Recent immigrant | Latin American | Middle | 77.47 | 56.01, 90.28 |  |
| 92 | Woman | Young adult | Recent immigrant | East Asian | Middle | 77.50 | 71.02, 82.88 |  |
| 93 | Woman | Middle-aged adult | Non-immigrant | East Asian | Low | 77.67 | 62.46, 87.91 |  |
| 94 | Man | Middle-aged adult | Non-immigrant | Latin American | Middle | 77.68 | 24.5, 97.39 |  |
| 95 | Woman | Young adult | Recent immigrant | South Asian | Low | 77.74 | 68.5, 84.86 |  |
| 96 | Man | Young adult | Non-immigrant | East Asian | High | 77.81 | 70.14, 83.96 |  |
| 97 | Woman | Young adult | Recent immigrant | White | High | 77.85 | 66.04, 86.4 |  |
| 98 | Woman | Young adult | Non-immigrant | South Asian | Middle | 78.38 | 66.22, 87.01 |  |
| 99 | Woman | Young adult | Non-immigrant | East Asian | Middle | 78.46 | 70.7, 84.61 |  |
| 100 | Woman | Young adult | Non-immigrant | West Asian | Middle | 78.48 | 61.85, 89.14 |  |
| 101 | Man | Older adult | Non-immigrant | East Asian | Low | 78.53 | 12.69, 98.93 |  |
| 102 | Woman | Young adult | Established immigrant | West Asian | Middle | 78.67 | 55.56, 91.58 |  |
| 103 | Woman | Middle-aged adult | Recent immigrant | White | Middle | 78.78 | 67.93, 86.67 |  |
| 104 | Woman | Middle-aged adult | Recent immigrant | East Asian | Low | 78.93 | 66.05, 87.82 |  |
| 105 | Woman | Older adult | Non-immigrant | East Asian | Low | 79.09 | 47.76, 94 |  |
| 106 | Woman | Young adult | Recent immigrant | Latin American | High | 79.32 | 22.96, 98.01 |  |
| 107 | Man | Middle-aged adult | Established immigrant | White | Low | 79.42 | 73.64, 84.2 |  |
| 108 | Woman | Young adult | Non-immigrant | East Asian | High | 79.52 | 69.4, 86.92 |  |
| 109 | Woman | Young adult | Recent immigrant | Latin American | Middle | 79.54 | 65.71, 88.75 |  |
| 110 | Woman | Young adult | Non-immigrant | White | Low | 79.73 | 78.27, 81.12 |  |
| 111 | Woman | Young adult | Established immigrant | Latin American | High | 79.90 | 27.52, 97.65 |  |
| 112 | Woman | Middle-aged adult | Non-immigrant | West Asian | Low | 79.94 | 7.98, 99.46 |  |
| 113 | Woman | Young adult | Established immigrant | Latin American | Low | 80.15 | 63.47, 90.36 |  |
| 114 | Man | Older adult | Recent immigrant | South Asian | Low | 80.22 | 19.13, 98.58 |  |
| 115 | Man | Middle-aged adult | Established immigrant | Latin American | Low | 80.33 | 62.4, 90.95 |  |
| 116 | Woman | Young adult | Established immigrant | White | High | 80.42 | 68.75, 88.46 |  |
| 117 | Woman | Young adult | Established immigrant | White | Middle | 81.02 | 74.19, 86.37 |  |
| 118 | Man | Young adult | Established immigrant | South Asian | Middle | 81.14 | 71.52, 88.05 |  |
| 119 | Woman | Young adult | Non-immigrant | Black | High | 81.31 | 62.32, 91.96 |  |
| 120 | Woman | Middle-aged adult | Recent immigrant | West Asian | Low | 81.46 | 68.7, 89.79 |  |
| 121 | Man | Middle-aged adult | Non-immigrant | East Asian | High | 81.47 | 58.52, 93.2 |  |
| 122 | Man | Older adult | Recent immigrant | West Asian | Low | 81.57 | 0.2, 99.99 |  |
| 123 | Woman | Middle-aged adult | Recent immigrant | East Asian | Middle | 81.77 | 71.1, 89.1 |  |
| 124 | Man | Middle-aged adult | Non-immigrant | East Asian | Low | 81.85 | 63.41, 92.15 |  |
| 125 | Man | Middle-aged adult | Non-immigrant | White | Middle | 82.01 | 80.81, 83.15 |  |
| 126 | Man | Middle-aged adult | Recent immigrant | South Asian | Low | 82.17 | 69.85, 90.16 |  |
| 127 | Man | Middle-aged adult | Recent immigrant | East Asian | Low | 82.17 | 73.62, 88.38 |  |
| 128 | Man | Young adult | Established immigrant | Black | High | 82.42 | 23.01, 98.66 |  |
| 129 | Woman | Middle-aged adult | Recent immigrant | West Asian | Middle | 82.49 | 27.12, 98.35 |  |
| 130 | Man | Middle-aged adult | Established immigrant | Black | Middle | 82.59 | 70.02, 90.59 |  |
| 131 | Woman | Young adult | Established immigrant | East Asian | Low | 82.63 | 75.92, 87.77 |  |
| 132 | Man | Middle-aged adult | Non-immigrant | West Asian | High | 82.96 | 0.54, 99.98 |  |
| 133 | Man | Middle-aged adult | Recent immigrant | West Asian | Middle | 83.12 | 51.3, 95.84 |  |
| 134 | Woman | Middle-aged adult | Non-immigrant | South Asian | Low | 83.33 | 30.04, 98.31 |  |
| 135 | Man | Middle-aged adult | Established immigrant | White | High | 83.37 | 78.92, 87.03 |  |
| 136 | Woman | Young adult | Non-immigrant | South Asian | High | 83.45 | 68.17, 92.23 |  |
| 137 | Man | Young adult | Established immigrant | West Asian | High | 83.48 | 54, 95.61 |  |
| 138 | Woman | Middle-aged adult | Recent immigrant | Latin American | Low | 83.73 | 66.32, 93.08 |  |
| 139 | Man | Older adult | Non-immigrant | West Asian | Low | 83.80 | 0.78, 99.97 |  |
| 140 | Woman | Young adult | Established immigrant | Black | Middle | 83.84 | 68.63, 92.49 |  |
| 141 | Woman | Young adult | Non-immigrant | Latin American | High | 83.92 | 47.86, 96.74 |  |
| 142 | Woman | Middle-aged adult | Recent immigrant | South Asian | Low | 84.20 | 61.05, 94.77 |  |
| 143 | Man | Middle-aged adult | Recent immigrant | West Asian | High | 84.50 | 4.13, 99.86 |  |
| 144 | Man | Middle-aged adult | Established immigrant | White | Middle | 84.52 | 79.45, 88.52 |  |
| 145 | Woman | Older adult | Recent immigrant | South Asian | High | 84.56 | 0.01, 100 |  |
| 146 | Woman | Young adult | Non-immigrant | West Asian | High | 84.56 | 12.49, 99.53 |  |
| 147 | Man | Older adult | Recent immigrant | Latin American | Low | 84.61 | 17.94, 99.28 |  |
| 148 | Man | Older adult | Recent immigrant | Black | Low | 84.69 | 29.21, 98.67 |  |
| 149 | Man | Middle-aged adult | Established immigrant | Latin American | Middle | 84.73 | 72.56, 92.09 |  |
| 150 | Woman | Middle-aged adult | Recent immigrant | East Asian | High | 84.88 | 72.08, 92.43 |  |
| 151 | Man | Middle-aged adult | Recent immigrant | East Asian | Middle | 84.97 | 74.06, 91.8 |  |
| 152 | Woman | Middle-aged adult | Non-immigrant | Latin American | High | 85.03 | 1.67, 99.95 |  |
| 153 | Man | Middle-aged adult | Recent immigrant | East Asian | High | 85.17 | 64.93, 94.68 |  |
| 154 | Woman | Young adult | Established immigrant | East Asian | Middle | 85.24 | 78.44, 90.17 |  |
| 155 | Woman | Middle-aged adult | Recent immigrant | White | High | 85.37 | 71.24, 93.22 |  |
| 156 | Man | Middle-aged adult | Recent immigrant | White | High | 85.41 | 75.8, 91.62 |  |
| 157 | Woman | Middle-aged adult | Recent immigrant | West Asian | High | 85.43 | 0.48, 99.99 |  |
| 158 | Woman | Middle-aged adult | Recent immigrant | Latin American | High | 85.49 | 3.69, 99.89 |  |
| 159 | Woman | Middle-aged adult | Recent immigrant | Latin American | Middle | 85.49 | 37.05, 98.33 |  |
| 160 | Woman | Middle-aged adult | Established immigrant | Black | Low | 85.59 | 76.14, 91.7 |  |
| 161 | Man | Young adult | Non-immigrant | Latin American | High | 85.59 | 16.23, 99.45 |  |
| 162 | Man | Older adult | Recent immigrant | White | Middle | 85.59 | 56.82, 96.41 |  |
| 163 | Man | Middle-aged adult | Non-immigrant | Latin American | High | 85.66 | 43.48, 97.89 |  |
| 164 | Woman | Older adult | Recent immigrant | East Asian | Middle | 85.86 | 61.13, 95.91 |  |
| 165 | Man | Young adult | Established immigrant | Latin American | Middle | 85.95 | 75.03, 92.57 |  |
| 166 | Woman | Young adult | Established immigrant | South Asian | Middle | 85.99 | 75.42, 92.47 |  |
| 167 | Woman | Young adult | Non-immigrant | White | Middle | 86.22 | 85.15, 87.21 |  |
| 168 | Woman | Middle-aged adult | Established immigrant | Latin American | Low | 86.24 | 74.45, 93.09 |  |
| 169 | Man | Older adult | Recent immigrant | White | Low | 86.41 | 2.29, 99.94 |  |
| 170 | Man | Young adult | Established immigrant | South Asian | Low | 86.56 | 72.51, 94.03 |  |
| 171 | Woman | Older adult | Recent immigrant | White | Middle | 86.66 | 40.1, 98.44 |  |
| 172 | Woman | Middle-aged adult | Established immigrant | White | Low | 86.75 | 81.28, 90.8 |  |
| 173 | Man | Older adult | Non-immigrant | Black | Low | 86.80 | 55.08, 97.24 |  |
| 174 | Woman | Middle-aged adult | Non-immigrant | White | Low | 86.80 | 85.38, 88.1 |  |
| 175 | Man | Middle-aged adult | Established immigrant | West Asian | High | 86.81 | 59.47, 96.72 |  |
| 176 | Man | Middle-aged adult | Established immigrant | Black | Low | 87.13 | 78.67, 92.55 |  |
| 177 | Man | Middle-aged adult | Established immigrant | East Asian | Low | 87.17 | 81.91, 91.07 |  |
| 178 | Man | Middle-aged adult | Non-immigrant | Black | Middle | 87.22 | 59.89, 96.9 |  |
| 179 | Woman | Middle-aged adult | Established immigrant | Latin American | Middle | 87.34 | 62.83, 96.57 |  |
| 180 | Man | Middle-aged adult | Established immigrant | Latin American | High | 87.37 | 59.13, 97.07 |  |
| 181 | Man | Middle-aged adult | Recent immigrant | South Asian | Middle | 87.47 | 67.06, 95.99 |  |
| 182 | Man | Middle-aged adult | Non-immigrant | South Asian | Middle | 87.48 | 62.69, 96.67 |  |
| 183 | Man | Middle-aged adult | Non-immigrant | South Asian | Low | 87.50 | 53.84, 97.67 |  |
| 184 | Woman | Older adult | Recent immigrant | East Asian | High | 87.56 | 22.26, 99.43 |  |
| 185 | Man | Middle-aged adult | Non-immigrant | White | High | 87.67 | 86.66, 88.61 |  |
| 186 | Woman | Young adult | Established immigrant | South Asian | High | 87.72 | 75.92, 94.18 |  |
| 187 | Woman | Young adult | Non-immigrant | White | High | 87.80 | 86.66, 88.86 |  |
| 188 | Woman | Middle-aged adult | Established immigrant | West Asian | Low | 87.91 | 76.8, 94.1 |  |
| 189 | Woman | Older adult | Recent immigrant | Black | Low | 87.98 | 6.27, 99.88 |  |
| 190 | Woman | Middle-aged adult | Non-immigrant | West Asian | Middle | 87.99 | 2.23, 99.96 |  |
| 191 | Woman | Young adult | Recent immigrant | South Asian | Middle | 88.08 | 81.69, 92.45 |  |
| 192 | Man | Older adult | Recent immigrant | Black | High | 88.15 | 0.12, 100 |  |
| 193 | Man | Older adult | Established immigrant | West Asian | Low | 88.27 | 72.86, 95.47 |  |
| 194 | Woman | Middle-aged adult | Non-immigrant | Latin American | Middle | 88.28 | 0.43, 99.99 |  |
| 195 | Woman | Middle-aged adult | Non-immigrant | East Asian | Middle | 88.37 | 79.52, 93.7 |  |
| 196 | Woman | Middle-aged adult | Established immigrant | East Asian | Low | 88.46 | 83.64, 92 |  |
| 197 | Man | Middle-aged adult | Recent immigrant | Latin American | High | 88.60 | 38.66, 98.97 |  |
| 198 | Woman | Middle-aged adult | Recent immigrant | Black | High | 88.79 | 80.42, 93.86 |  |
| 199 | Man | Older adult | Recent immigrant | Latin American | Middle | 88.83 | 47.77, 98.57 |  |
| 200 | Man | Older adult | Established immigrant | Latin American | Low | 88.84 | 71.71, 96.15 |  |
| 201 | Man | Middle-aged adult | Established immigrant | West Asian | Middle | 88.90 | 78.98, 94.46 |  |
| 202 | Man | Older adult | Recent immigrant | Black | Middle | 89.04 | 55.14, 98.17 |  |
| 203 | Woman | Middle-aged adult | Non-immigrant | Black | Middle | 89.10 | 2.49, 99.96 |  |
| 204 | Woman | Young adult | Established immigrant | South Asian | Low | 89.19 | 81.79, 93.81 |  |
| 205 | Woman | Middle-aged adult | Established immigrant | West Asian | Middle | 89.23 | 74.3, 95.96 |  |
| 206 | Woman | Young adult | Established immigrant | Black | High | 89.24 | 0.69, 99.99 |  |
| 207 | Woman | Older adult | Non-immigrant | Latin American | Middle | 89.51 | 0, 100 |  |
| 208 | Woman | Older adult | Recent immigrant | Latin American | Low | 89.60 | 33.37, 99.33 |  |
| 209 | Man | Older adult | Recent immigrant | West Asian | Middle | 89.69 | 44.53, 98.95 |  |
| 210 | Man | Older adult | Recent immigrant | West Asian | High | 89.78 | 53.43, 98.54 |  |
| 211 | Woman | Young adult | Established immigrant | West Asian | High | 89.90 | 18.63, 99.71 |  |
| 212 | Woman | Older adult | Recent immigrant | White | Low | 89.91 | 10.21, 99.86 |  |
| 213 | Man | Older adult | Non-immigrant | White | Low | 89.93 | 89.15, 90.67 |  |
| 214 | Man | Older adult | Non-immigrant | Black | High | 90.13 | 4.97, 99.94 |  |
| 215 | Woman | Young adult | Established immigrant | East Asian | High | 90.14 | 82.73, 94.58 |  |
| 216 | Woman | Older adult | Non-immigrant | Black | Low | 90.29 | 55.56, 98.57 |  |
| 217 | Woman | Middle-aged adult | Non-immigrant | West Asian | High | 90.31 | 59.47, 98.34 |  |
| 218 | Man | Middle-aged adult | Established immigrant | South Asian | Middle | 90.39 | 83.72, 94.51 |  |
| 219 | Man | Older adult | Non-immigrant | West Asian | Middle | 90.41 | 0.34, 100 |  |
| 220 | Woman | Middle-aged adult | Non-immigrant | White | Middle | 90.62 | 89.7, 91.46 |  |
| 221 | Man | Middle-aged adult | Established immigrant | East Asian | High | 90.62 | 84.5, 94.48 |  |
| 222 | Woman | Middle-aged adult | Non-immigrant | Latin American | Low | 90.72 | 78.75, 96.27 |  |
| 223 | Man | Older adult | Established immigrant | White | Low | 90.77 | 88.39, 92.71 |  |
| 224 | Woman | Older adult | Non-immigrant | West Asian | Low | 90.84 | 69.37, 97.75 |  |
| 225 | Woman | Older adult | Established immigrant | West Asian | Low | 90.95 | 56.39, 98.73 |  |
| 226 | Man | Older adult | Recent immigrant | East Asian | Low | 90.97 | 17.78, 99.79 |  |
| 227 | Woman | Middle-aged adult | Recent immigrant | South Asian | Middle | 91.03 | 80.28, 96.2 |  |
| 228 | Man | Older adult | Recent immigrant | East Asian | Middle | 91.14 | 36.82, 99.45 |  |
| 229 | Woman | Middle-aged adult | Non-immigrant | South Asian | Middle | 91.17 | 5.6, 99.94 |  |
| 230 | Man | Middle-aged adult | Established immigrant | East Asian | Middle | 91.20 | 85.91, 94.63 |  |
| 231 | Woman | Older adult | Non-immigrant | White | Low | 91.36 | 90.45, 92.2 |  |
| 232 | Man | Middle-aged adult | Recent immigrant | South Asian | High | 91.37 | 8.89, 99.91 |  |
| 233 | Man | Older adult | Non-immigrant | Black | Middle | 91.49 | 85.78, 95.04 |  |
| 234 | Woman | Older adult | Non-immigrant | Latin American | Low | 91.56 | 62.19, 98.62 |  |
| 235 | Man | Older adult | Recent immigrant | East Asian | High | 91.56 | 45.7, 99.29 |  |
| 236 | Man | Middle-aged adult | Established immigrant | South Asian | High | 91.57 | 83.73, 95.82 |  |
| 237 | Woman | Middle-aged adult | Established immigrant | White | Middle | 91.58 | 88.85, 93.69 |  |
| 238 | Woman | Middle-aged adult | Non-immigrant | Black | High | 91.61 | 51.18, 99.13 |  |
| 239 | Man | Older adult | Non-immigrant | South Asian | Low | 91.65 | 60.31, 98.75 |  |
| 240 | Man | Older adult | Non-immigrant | West Asian | High | 91.91 | 13.02, 99.88 |  |
| 241 | Man | Older adult | Established immigrant | East Asian | Low | 91.96 | 86.78, 95.22 |  |
| 242 | Woman | Middle-aged adult | Established immigrant | Latin American | High | 91.96 | 69.51, 98.29 |  |
| 243 | Woman | Middle-aged adult | Established immigrant | Black | Middle | 92.07 | 68.94, 98.38 |  |
| 244 | Woman | Older adult | Established immigrant | White | Low | 92.44 | 90.37, 94.09 |  |
| 245 | Man | Older adult | Non-immigrant | Latin American | High | 92.45 | 56.1, 99.15 |  |
| 246 | Woman | Middle-aged adult | Established immigrant | White | High | 92.58 | 89.33, 94.89 |  |
| 247 | Man | Older adult | Recent immigrant | White | High | 92.62 | 86.07, 96.23 |  |
| 248 | Woman | Middle-aged adult | Non-immigrant | White | High | 92.67 | 91.96, 93.32 |  |
| 249 | Woman | Older adult | Recent immigrant | Latin American | Middle | 92.71 | 69.29, 98.62 |  |
| 250 | Woman | Middle-aged adult | Established immigrant | East Asian | Middle | 92.75 | 89.42, 95.08 |  |
| 251 | Woman | Older adult | Recent immigrant | East Asian | Low | 92.88 | 25.03, 99.8 |  |
| 252 | Woman | Older adult | Recent immigrant | Black | Middle | 92.91 | 2.03, 99.99 |  |
| 253 | Man | Middle-aged adult | Non-immigrant | South Asian | High | 92.94 | 19.8, 99.86 |  |
| 254 | Woman | Older adult | Established immigrant | Black | Middle | 92.97 | 78.59, 97.94 |  |
| 255 | Woman | Older adult | Recent immigrant | West Asian | High | 93.04 | 61.84, 99.1 |  |
| 256 | Woman | Older adult | Non-immigrant | West Asian | Middle | 93.08 | 66.94, 98.89 |  |
| 257 | Woman | Middle-aged adult | Established immigrant | East Asian | High | 93.09 | 86.96, 96.46 |  |
| 258 | Woman | Older adult | Non-immigrant | South Asian | Low | 93.14 | 6.19, 99.96 |  |
| 259 | Woman | Older adult | Recent immigrant | West Asian | Middle | 93.16 | 83.04, 97.43 |  |
| 260 | Woman | Older adult | Non-immigrant | East Asian | Middle | 93.23 | 73.29, 98.57 |  |
| 261 | Woman | Older adult | Recent immigrant | White | High | 93.43 | 9.49, 99.95 |  |
| 262 | Man | Older adult | Established immigrant | Black | Low | 93.45 | 82.96, 97.66 |  |
| 263 | Man | Middle-aged adult | Established immigrant | Black | High | 93.45 | 82.89, 97.68 |  |
| 264 | Man | Older adult | Non-immigrant | White | Middle | 93.54 | 92.9, 94.12 |  |
| 265 | Man | Older adult | Non-immigrant | White | High | 93.55 | 92.7, 94.31 |  |
| 266 | Woman | Older adult | Non-immigrant | East Asian | High | 93.80 | 77.47, 98.52 |  |
| 267 | Woman | Older adult | Recent immigrant | Latin American | High | 93.81 | 67.82, 99.09 |  |
| 268 | Woman | Older adult | Established immigrant | South Asian | Middle | 93.86 | 86.99, 97.22 |  |
| 269 | Man | Older adult | Non-immigrant | South Asian | Middle | 93.95 | 77.86, 98.56 |  |
| 270 | Man | Older adult | Established immigrant | Black | High | 94.01 | 42.79, 99.7 |  |
| 271 | Man | Older adult | Established immigrant | West Asian | Middle | 94.15 | 49.94, 99.62 |  |
| 272 | Woman | Older adult | Non-immigrant | Black | High | 94.18 | 85.25, 97.84 |  |
| 273 | Woman | Older adult | Non-immigrant | White | Middle | 94.19 | 93.67, 94.66 |  |
| 274 | Man | Older adult | Non-immigrant | East Asian | Middle | 94.21 | 3.78, 99.99 |  |
| 275 | Woman | Older adult | Non-immigrant | West Asian | High | 94.25 | 80.94, 98.44 |  |
| 276 | Woman | Older adult | Established immigrant | Black | Low | 94.26 | 87.73, 97.42 |  |
| 277 | Woman | Middle-aged adult | Recent immigrant | South Asian | High | 94.28 | 4.21, 99.98 |  |
| 278 | Man | Older adult | Established immigrant | Black | Middle | 94.34 | 81.93, 98.39 |  |
| 279 | Woman | Middle-aged adult | Established immigrant | South Asian | High | 94.40 | 43.47, 99.73 |  |
| 280 | Woman | Middle-aged adult | Non-immigrant | South Asian | High | 94.40 | 26.83, 99.87 |  |
| 281 | Woman | Older adult | Established immigrant | West Asian | Middle | 94.41 | 6.42, 99.98 |  |
| 282 | Woman | Middle-aged adult | Non-immigrant | East Asian | High | 94.41 | 83.29, 98.28 |  |
| 283 | Man | Older adult | Established immigrant | Latin American | Middle | 94.44 | 26.32, 99.88 |  |
| 284 | Man | Older adult | Recent immigrant | South Asian | High | 94.50 | 87, 97.78 |  |
| 285 | Man | Older adult | Established immigrant | White | High | 94.51 | 92.63, 95.93 |  |
| 286 | Woman | Older adult | Non-immigrant | Black | Middle | 94.54 | 90.44, 96.94 |  |
| 287 | Man | Older adult | Recent immigrant | South Asian | Middle | 94.58 | 4.11, 99.99 |  |
| 288 | Man | Older adult | Established immigrant | White | Middle | 94.61 | 92.74, 96.02 |  |
| 289 | Woman | Older adult | Established immigrant | White | Middle | 94.63 | 92.1, 96.38 |  |
| 290 | Woman | Older adult | Non-immigrant | Latin American | High | 94.68 | 85.72, 98.14 |  |
| 291 | Man | Older adult | Established immigrant | South Asian | Low | 94.76 | 85.34, 98.25 |  |
| 292 | Woman | Older adult | Established immigrant | East Asian | Middle | 94.90 | 90.36, 97.37 |  |
| 293 | Woman | Older adult | Established immigrant | Latin American | Low | 94.91 | 52.69, 99.68 |  |
| 294 | Man | Older adult | Non-immigrant | South Asian | High | 94.91 | 72.24, 99.26 |  |
| 295 | Woman | Middle-aged adult | Established immigrant | Black | High | 94.97 | 28.11, 99.89 |  |
| 296 | Man | Older adult | Established immigrant | Latin American | High | 95.11 | 4.22, 99.99 |  |
| 297 | Woman | Middle-aged adult | Established immigrant | West Asian | High | 95.14 | 41.09, 99.82 |  |
| 298 | Woman | Older adult | Non-immigrant | South Asian | Middle | 95.50 | 2.23, 99.99 |  |
| 299 | Woman | Older adult | Established immigrant | East Asian | Low | 95.52 | 91.37, 97.73 |  |
| 300 | Man | Older adult | Established immigrant | South Asian | Middle | 95.62 | 88.88, 98.35 |  |
| 301 | Man | Older adult | Established immigrant | West Asian | High | 95.63 | 92.66, 97.43 |  |
| 302 | Woman | Older adult | Recent immigrant | South Asian | Middle | 95.66 | 90.82, 98 |  |
| 303 | Man | Older adult | Non-immigrant | East Asian | High | 95.67 | 93.19, 97.27 |  |
| 304 | Woman | Older adult | Non-immigrant | White | High | 95.74 | 95.12, 96.28 |  |
| 305 | Woman | Older adult | Recent immigrant | South Asian | Low | 95.76 | 26.48, 99.93 |  |
| 306 | Man | Middle-aged adult | Established immigrant | South Asian | Low | 95.79 | 91.03, 98.07 |  |
| 307 | Woman | Middle-aged adult | Established immigrant | South Asian | Middle | 95.94 | 91.24, 98.17 |  |
| 308 | Woman | Older adult | Established immigrant | White | High | 96.10 | 93.8, 97.57 |  |
| 309 | Woman | Older adult | Non-immigrant | South Asian | High | 96.15 | 9.3, 99.98 |  |
| 310 | Man | Older adult | Established immigrant | East Asian | Middle | 96.18 | 93.31, 97.85 |  |
| 311 | Man | Older adult | Established immigrant | South Asian | High | 96.21 | 82.53, 99.27 |  |
| 312 | Woman | Middle-aged adult | Established immigrant | South Asian | Low | 96.36 | 90.86, 98.6 |  |
| 313 | Woman | Older adult | Established immigrant | Latin American | Middle | 96.41 | 47.76, 99.87 |  |
| 314 | Man | Older adult | Established immigrant | East Asian | High | 96.63 | 92.61, 98.49 |  |
| 315 | Woman | Older adult | Established immigrant | West Asian | High | 96.77 | 94.24, 98.21 |  |
| 316 | Woman | Older adult | Established immigrant | Black | High | 96.91 | 95.05, 98.09 |  |
| 317 | Woman | Older adult | Established immigrant | Latin American | High | 97.27 | 94.38, 98.69 |  |
| 318 | Woman | Older adult | Established immigrant | East Asian | High | 97.45 | 91.86, 99.23 |  |
| 319 | Woman | Older adult | Established immigrant | South Asian | Low | 97.79 | 72.57, 99.86 |  |
| 320 | Woman | Older adult | Established immigrant | South Asian | High | 98.44 | 50.92, 99.97 |  |

|  | Gender | Age | Immigration status | Race | Income | 95% CI |
| --- | --- | --- | --- | --- | --- | --- |
| 1 | Woman | Older adult | Non-immigrant | Latin American | Middle | 00.00, 100.0 |
| 2 | Woman | Older adult | Recent immigrant | South Asian | High | 00.01, 100.0 |
| 3 | Man | Middle-aged adult | Non-immigrant | West Asian | Middle | 00.10, 99.99 |
| 4 | Man | Older adult | Recent immigrant | Black | High | 00.12, 100.0 |
| 5 | Man | Older adult | Recent immigrant | West Asian | Low | 00.20, 99.99 |
| 6 | Man | Older adult | Non-immigrant | West Asian | Middle | 00.34, 100.0 |
| 7 | Woman | Middle-aged adult | Non-immigrant | Latin American | Middle | 00.43, 99.99 |
| 8 | Woman | Middle-aged adult | Recent immigrant | West Asian | High | 00.48, 99.99 |
| 9 | Man | Middle-aged adult | Non-immigrant | West Asian | High | 00.54, 99.98 |
| 10 | Woman | Young adult | Established immigrant | Black | High | 00.69, 99.99 |
| 11 | Man | Older adult | Non-immigrant | West Asian | Low | 00.78, 99.97 |
| 12 | Woman | Middle-aged adult | Non-immigrant | Latin American | High | 01.67, 99.95 |
| 13 | Woman | Older adult | Recent immigrant | Black | Middle | 02.03, 99.99 |
| 14 | Woman | Older adult | Non-immigrant | South Asian | Middle | 02.23, 99.99 |
| 15 | Woman | Middle-aged adult | Non-immigrant | West Asian | Middle | 02.23, 99.96 |
| 16 | Man | Older adult | Recent immigrant | White | Low | 02.29, 99.94 |
| 17 | Woman | Middle-aged adult | Non-immigrant | Black | Middle | 02.49, 99.96 |
| 18 | Woman | Older adult | Recent immigrant | West Asian | Low | 02.90, 99.74 |
| 19 | Man | Older adult | Non-immigrant | East Asian | Middle | 03.78, 99.99 |
| 20 | Woman | Middle-aged adult | Recent immigrant | Latin American | High | 03.69, 99.89 |
| 21 | Man | Older adult | Recent immigrant | South Asian | Middle | 04.11, 99.99 |
| 22 | Woman | Middle-aged adult | Recent immigrant | South Asian | High | 04.21, 99.98 |
| 23 | Man | Older adult | Established immigrant | Latin American | High | 04.22, 99.99 |
| 24 | Man | Middle-aged adult | Recent immigrant | West Asian | High | 04.13, 99.86 |
| 25 | Man | Older adult | Non-immigrant | Black | High | 04.97, 99.94 |
| 26 | Woman | Middle-aged adult | Non-immigrant | South Asian | Middle | 05.60, 99.94 |
| 27 | Woman | Older adult | Non-immigrant | South Asian | Low | 06.19, 99.96 |
| 28 | Woman | Older adult | Recent immigrant | Black | Low | 06.27, 99.88 |
| 29 | Woman | Older adult | Established immigrant | West Asian | Middle | 06.42, 99.98 |
| 30 | Woman | Middle-aged adult | Non-immigrant | West Asian | Low | 07.98, 99.46 |
| 31 | Man | Middle-aged adult | Recent immigrant | South Asian | High | 08.89, 99.91 |
| 32 | Man | Middle-aged adult | Non-immigrant | West Asian | Low | 08.32, 99.13 |

Table B. Intersections with the widest (10%) predicted probability confidence intervals

|  | Gender | Age | Immigration status | Race | Income | 95% CI |
| --- | --- | --- | --- | --- | --- | --- |
| 1 | Woman | Middle-aged adult | Established immigrant | White | High | 89.33, 94.89 |
| 2 | Woman | Middle-aged adult | Established immigrant | White | Middle | 88.85, 93.69 |
| 3 | Man | Older adult | Established immigrant | West Asian | High | 92.66, 97.43 |
| 4 | Man | Older adult | Established immigrant | East Asian | Middle | 93.31, 97.85 |
| 5 | Man | Older adult | Established immigrant | White | Low | 88.39, 92.71 |
| 6 | Woman | Older adult | Established immigrant | Latin American | High | 94.38, 98.69 |
| 7 | Woman | Older adult | Established immigrant | White | Middle | 92.10, 96.38 |
| 8 | Man | Older adult | Non-immigrant | East Asian | High | 93.19, 97.27 |
| 9 | Man | Young adult | Non-immigrant | White | Low | 62.27, 66.26 |
| 10 | Woman | Older adult | Established immigrant | West Asian | High | 94.24, 98.21 |
| 11 | Man | Middle-aged adult | Non-immigrant | White | Low | 74.25, 78.19 |
| 12 | Woman | Older adult | Established immigrant | White | High | 93.80, 97.57 |
| 13 | Woman | Older adult | Established immigrant | White | Low | 90.37, 94.09 |
| 14 | Man | Older adult | Established immigrant | White | High | 92.63, 95.93 |
| 15 | Man | Older adult | Established immigrant | White | Middle | 92.74, 96.02 |
| 16 | Woman | Older adult | Established immigrant | Black | High | 95.05, 98.09 |
| 17 | Woman | Young adult | Non-immigrant | White | Low | 78.27, 81.12 |
| 18 | Man | Young adult | Non-immigrant | White | Middle | 69.21, 71.99 |
| 19 | Woman | Middle-aged adult | Non-immigrant | White | Low | 85.38, 88.10 |
| 20 | Man | Young adult | Non-immigrant | White | High | 72.79, 75.49 |
| 21 | Man | Middle-aged adult | Non-immigrant | White | Middle | 80.81, 83.15 |
| 22 | Woman | Young adult | Non-immigrant | White | High | 86.66, 88.86 |
| 23 | Woman | Young adult | Non-immigrant | White | Middle | 85.15, 87.21 |
| 24 | Man | Middle-aged adult | Non-immigrant | White | High | 86.66, 88.61 |
| 25 | Woman | Middle-aged adult | Non-immigrant | White | Middle | 89.70, 91.46 |
| 26 | Woman | Older adult | Non-immigrant | White | Low | 90.45, 92.20 |
| 27 | Man | Older adult | Non-immigrant | White | High | 92.70, 94.31 |
| 28 | Man | Older adult | Non-immigrant | White | Low | 89.15, 90.67 |
| 29 | Woman | Middle-aged adult | Non-immigrant | White | High | 91.96, 93.32 |
| 30 | Man | Older adult | Non-immigrant | White | Middle | 92.90, 94.12 |
| 31 | Woman | Older adult | Non-immigrant | White | High | 95.12, 96.28 |
| 32 | Woman | Older adult | Non-immigrant | White | Middle | 93.67, 94.66 |

Table C. Intersections with the narrowest (10%) predicted probability confidence intervals
